# *In vivo* analysis of formation and endocytosis of the Wnt/β-Catenin signaling complex in zebrafish embryos

**DOI:** 10.1242/jcs.148767

**Published:** 2014-09-15

**Authors:** Anja I. H. Hagemann, Jennifer Kurz, Silke Kauffeld, Qing Chen, Patrick M. Reeves, Sabrina Weber, Simone Schindler, Gary Davidson, Tomas Kirchhausen, Steffen Scholpp

**Affiliations:** 1 Karlsruhe Institute of Technology (KIT), Institute of Toxicology and Genetics (ITG), 76021 Karsruhe, Germany; 2 Departments of Cell Biology and Pediatrics, Harvard Medical School and Program in Cellular and Molecular Medicine at Boston Children's Hospital, Boston, 02115 MA, USA

**Keywords:** Wnt signaling, Development, Endocytosis, Signalosome, Zebrafish

## Abstract

After activation by Wnt/β-Catenin ligands, a multi-protein complex assembles at the clustering membrane-bound receptors and intracellular signal transducers into the so-called Lrp6-signalosome. However, the mechanism of signalosome formation and dissolution is yet not clear. Our imaging studies of live zebrafish embryos show that the signalosome is a highly dynamic structure. It is continuously assembled by Dvl2-mediated recruitment of the transducer complex to the activated receptors and partially disassembled by endocytosis. We find that, after internalization, the ligand-receptor complex and the transducer complex take separate routes. The Wnt–Fz–Lrp6 complex follows a Rab-positive endocytic path. However, when still bound to the transducer complex, Dvl2 forms intracellular aggregates. We show that this endocytic process is not only essential for ligand-receptor internalization but also for signaling. The μ2-subunit of the endocytic Clathrin adaptor Ap2 interacts with Dvl2 to maintain its stability during endocytosis. Blockage of Ap2μ2 function leads to Dvl2 degradation, inhibiton of signalosome formation at the plasma membrane and, consequently, reduction of signaling. We conclude that Ap2μ2-mediated endocytosis is important to maintain Wnt/β-catenin signaling in vertebrates.

## INTRODUCTION

Wnt/β-Catenin signaling is an essential, evolutionarily conserved pathway in embryonic development, stem cell biology and human disease, including cancer (Angers and Moon, 2009; Clevers and Nusse, 2012). In the Wnt-off state, β-Catenin is constantly targeted for degradation by a multi-protein destruction complex containing Axin1, adenomatous polyposis coli (APC), Casein Kinase 1α (CK1α) and Glycogen Synthase Kinase 3β (GSK3β). GSK3β phosphorylates β-Catenin priming it for ubiquitination by the E3 ubiquitin ligase beta-transducing repeat-containing protein (β-TrCP) and subsequent proteasomal degradation. Upon Wnt ligand stimulation, Wnt receptor Frizzled (Fz) and the low-density lipoprotein receptor-related proteins 5 and 6 (Lrp5 and Lrp6; hereafter referred to as Lrp5/6) cluster together. This ligand-receptor complex recruits cytoplasmic proteins such as Dvl and Axin1 to the membrane (Cong et al., 2004). Fz binds to Dvl, and Lrp5/6 bind to Axin1 and GSK3β. The ligand-receptor complex and the transducer complex form a membranous multi-protein complex congruously termed Lrp6-signalosome (Bilic et al., 2007), which inhibits the kinase activity of GSK3β by competitively binding it to Lrp5/6. However, the necessity of ligand-receptor internalization for signal maintenance is controversially discussed (Kim et al., 2013b; Metcalfe et al., 2010; Seto and Bellen, 2006). Recently, it was proposed that the destruction complex is non-disintegrating throughout the entire cycle of signal activation and inhibition. In this study in human cell culture, however, there was no indication found for endocytosis of an activated ligand-receptor complex (Li et al., 2012). In contrast, in an alternative model it was suggested that all signaling components, including Wnt, become sequestered in late endosomes or multi-vesicular bodies (MVBs) to maintain signaling (Dobrowolski et al., 2012; Taelman et al., 2010). Furthermore, there is an on-going discussion about the type of endocytosis required for Wnt/β-Catenin signaling. It has been suggested that Caveolin-dependent endocytosis is required for Lrp6 activity (Bilic et al., 2007; Demir et al., 2013; Glinka et al., 2011; Ohkawara et al., 2011; Yamamoto et al., 2006) whereas it has also proposed by others that Clathrin-mediated endocytosis (CME) is important for Wnt/β-Catenin signaling (Blitzer and Nusse, 2006; Kikuchi et al., 2009). In addition, CME has also been found to regulate non-canonical Wnt/planar cell polarity (Wnt/PCP) signaling (Chen et al., 2003; Kim et al., 2008; Yu et al., 2007).

During the endocytic process, Clathrin does not bind directly to the membrane or to cargo receptors but relies on linker complexes such as the adaptor protein 2 (Ap2). This Clathrin adaptor is a heterotetramer with the four subunits α1, β1, μ2 and σ1 (Kirchhausen, 2012; Traub, 2009). It links Clathrin to the cytoplasmic membrane, and is responsible for cargo selection and recruitment into forming coated pits. In fact, the cargo binding subunit Ap2μ2 was shown to bind to Dvl2, which – in turn – recruits Fz4 for CME, which is essential for Wnt/PCP signaling during *Xenopus* gastrulation (Yu et al., 2007). Since Dvl2 is the common hub protein of Wnt/PCP and Wnt/β-Catenin signaling, we reasoned that functional analysis of its postulated binding partner Ap2μ2 would allow us to clarify the necessity of endocytosis for Wnt/β-Catenin signaling. To dissect the requirement of Ap2μ2-mediated endocytosis in canonical and non-canonical Wnt signaling in a functional tissue, we chose zebrafish embryos at blastula stage as these provide an optimal *in vivo* system to dissect both Wnt signaling pathways in an intact organism. At this stage, Wnt/β-Catenin signaling is already detectable, whereas Wnt/PCP signaling becomes active only during late gastrulation (Ulrich et al., 2003).

We find that endocytosis has an essential function during the process of Wnt/β-Catenin signal transduction in vertebrates. Overexpression of the Ap2μ2 subunit enhances the expression of direct Wnt/β-Catenin target genes and knockdown of the subunit decreases their expression. By using live confocal imaging analysis of intact zebrafish embryos, we show that Wnt8-induced Lrp6-signalosome formation requires the interaction of Ap2μ2 and Dvl2 at the plasma membrane. Wnt8, subsequently, becomes internalized in a non-cell autonomous fashion together with the receptors and the intracellular signal transducers. Along the endocytic route, however, their paths are quickly separated. We detect colocalization of the ligand-receptor complex by using endocytic Rab protein markers, such as Rab5 and Rab7, whereas the transducer complex, composed of Dvl2, Axin1, GSK3β and β-Catenin, forms independent cytoplasmic aggregates. Hence, we conclude that the transducer complex is dissociated from the vesicular membrane early after internalization. In summary, we show that interaction between Dvl2 and Ap2μ2 is required and sufficient for the formation and endocytosis of the signalosome including the dissociation of the transducer complex from the ligand-receptor complex *in vivo*. During endocytosis, Ap2μ2 protects Dvl2 from degradation to maintain signaling. Thus Ap2μ2 mediated endocytosis of the Lrp6-signalosome is an important step for Wnt/β-Catenin signaling maintenance in vertebrates.

## RESULTS

### Ap2μ2 is essential for Wnt/β-Catenin and PCP signaling in early zebrafish embryos

**Fig. 1.**
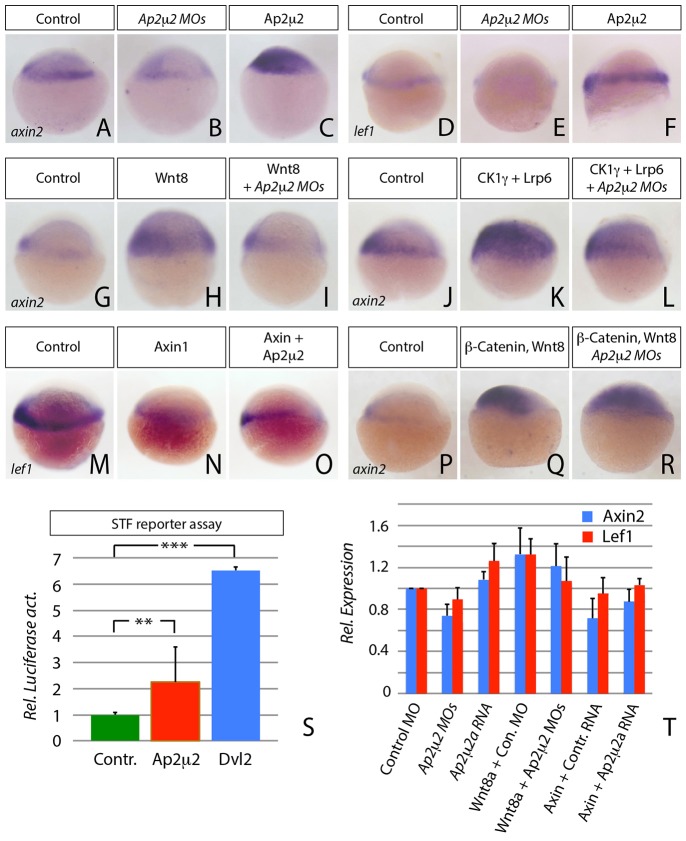
**AP2μ2 has an essential function in Wnt/β-Catenin signaling.** (A–F and H–S) *In situ* hybridization against *lef1* or *axin2* at shield stage. Embryos were injected with indicated mRNAs or morpholinos (MOs) and the lateral view is shown ( the dorsal side is on the left). (A–F) Expression of the Wnt target genes *axin2* and *lef1* is upregulated after Ap2μ2 protein synthesis, whereas the expression of both genes is downregulated when Ap2μ2 function is blocked. (A) *n* = 33; (B) *n* = 15/21; (C) *n* = 19/20; (D) *n* = 38; (E) 9/13; (F) 15/15. (G–L) Blockage of Ap2μ2 function by injection of 0.5 mM *ap2μ2a/b* morpholino solution rescues ectopic activation of indicated Wnt target gene expression by 1 ng mRNA of *Wnt8*, *Ck1γ*, and 2 ng *Lrp6*. (G) *n* = 29; (H) *n* = 20/28; (I) *n* = 13/21; (J) *n* = 45; (K) *n* = 21/22; (L) *n* = 17/22. (M–O) Overexpression of 2 ng *Ap2μ2a* mRNA counteracts the downregulation of *lef1* after injection of 2 ng of axin1 mRNA. (P–R) Injection of *ap2μ2a/b* double morpholino oligomeres did not alter the expression of axin2 after Wnt-activation by 1 ng of β-Catenin mRNA. (M) *n* = 37; (N) *n* = 25/26; (O) *n* = 20/23; (P) *n* = 44; (Q) *n* = 23/28; (R) *n* = 13/16. (S) Luciferase assay with STF reporter construct in shield-stage zebrafish embryos. Error bars are given as +s.e. from four independent measurements. For each measurement ten embryos were pooled. **P<0.005, ***P<0.001. (T) Relative target gene expression analyzed by qRT-PCR with primers against *axin2* (blue) and *lef1* (red) on embryonic cDNA injected with the indicated constructs. Error bars are given as +s.e. of two independent experiments scanned each in duplicates.

In mice, targeted gene disruption of the subunit Ap2μ2 is lethal before day 3,5 pc (Mitsunari et al., 2005). Therefore, we used a morpholino-based antisense approach to gradually knock-down Ap2μ2 function in zebrafish at blastula stage, where there are two homologues expressed with redundant function. Analysis of the efficiency of the morpholino oligomers, validation of their specificity by rescue experiments and detailed description of the late morphological phenotype of embryos with altered Ap2μ2 levels were carried out (described in supplementary material Figs S1, S2) In Ap2μ2a/μ2b double morphant embryos, we found that expression of bona-fide Wnt/β-Catenin target genes such as *axin2* and *lef1* was reduced (Fig. 1A,B and D,E) while overexpression of Ap2μ2a (hereafter Ap2μ2) enhanced Wnt/β-Catenin signaling in a concentration dependent manner (Fig. 1C,F and supplementary material Fig. S2A-D). We confirmed Wnt signaling activity of Ap2μ2 by real-time quantitative PCR (RT-qPCR) for induction of *axin2* and *lef1* expression (Fig. 1T) and in a luciferase-based Wnt reporter assay with SuperTopFlash (STF) reporter construct where it led to transcription activity in zebrafish embryos that was enhanced 2.2-fold (Fig. 1S). In the same luciferase assay, Dvl2 overexpression led to a 6.5-fold enhancement of transcriptional activation. This suggests that Ap2μ2 has an essential function during Wnt/β-Catenin signaling in blastula-stage embryos.

### Ap2μ2 is required downstream of Wnt, Lrp6 and Axin, but upstream of β-Catenin

To determine the level on which Ap2μ2 acts in the Wnt/β-Catenin signaling pathway, we analyzed its epistasis effect in the context of other members of the pathway. We used Wnt8 (i.e. open reading frame 1 of the zebrafish Wnt8a, Wnt8a-ORF1) for the analysis of canonical Wnt signaling because it is already expressed at 30% epiboly and, therefore, the first-acting Wnt/β-Catenin ligand during zebrafish development (Kelly et al., 1995). We found, that the activation of target gene expression by Wnt8 was counteracted by Ap2μ2 knockdown (Fig. 1H-J,V). Similarly the activation of the Wnt/β-Catenin pathway by overexpression of the co-receptor Lrp6 and CK1γ, a membrane-tethered kinase of Lrp6, could be repressed by knockdown of Ap2μ2 (Fig. 1K-M). Consistently, the inhibitory effect of Axin1, a member of the β-Catenin destruction complex, was prevented by Ap2μ2 overexpression (Fig. 1N-P,V). Induction of *axin2* expression by overexpressed β-Catenin, however, was not impaired in Ap2μ2 morphant embryos (Fig. 1Q-S). Hence, we conclude that Ap2μ2 function is required downstream or in parallel of the Lrp6-signalosome and upstream of β-Catenin.

### Wnt8 induced signalosome formation at the plasma membrane is dependent on Ap2μ2 function

So far, the Lrp6-signalosome has been proposed to be necessary for Lrp6 phosphorylation and signaling in cell culture (Bilic et al., 2007). However, the significance of signalosomes and their internalization in living organisms has so far not been established. Therefore, we asked whether Wnt stimulation is able to induce signalosome formation at the plasma membrane of zebrafish blastula-stage embryos and whether such signalosomes would become internalized.

**Fig. 2.**
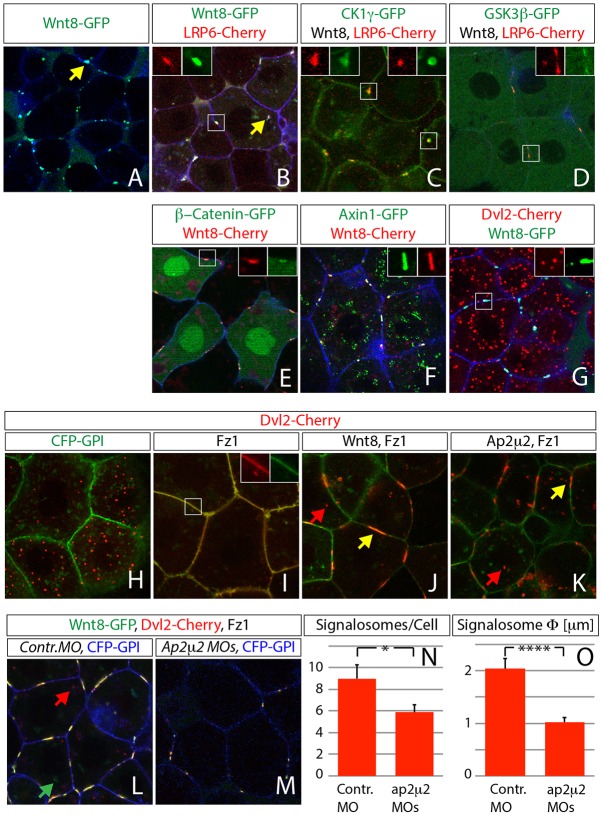
**Signalosome formation at the cytoplasmic membrane is dependent on Ap2μ2 function.** Confocal microscopy analysis of live zebrafish embryos that express the indicated constructs at 30–50% epiboly stage; all constructs were used at 1 ng mRNA, except Axin1 and Dvl2 (used at 2 ng) and Ap2μ2 (used at 1,6 ng). Confocal images represent single *z*-sections. Constructs were expressed as indicated and are shown in the indicated colors together with 1,2 ng mRNA of the membrane marker CFP-GPI (blue in A,B,D,E–G,L,M). Magnified images in B-G and I represent boxed areas with split channels related to the indicated color code for injected constructs. Expression of Wnt8-GFP mRNA lead to the formation of signalosomes positive for Lrp6, CK1γ, β-Catenin, GSK3β, Axin1 and Dvl2 (A–D, J,L,M). Single channels for images B-G are shown in supplementary material Fig. S3 and control images of the indicated expression constructs in supplementary material Fig. S4. (H) Control of Dvl2 with CFPGPI (green). (I) Fz1 co-expression recruits Dvl2 to the plasma membrane. (J–L) Formation of the signalosome can be induced by expression of Wnt8 mRNA or Ap2μ2 mRNA (K). (L,M) Injection of 0.5 mM morpholino (MO) oligomers targeting Ap2μ2a and Ap2μ2b leads to strong reduction of Wnt8- and Dvl2-positive signalosomes at the plasma membrane. Yellow arrow, signalosome; green arrow, intracellular Wnt8 puncta; red arrow, intracellular Dvl2 puncta. (N,O) Manual quantification and measurment of signalosomes at the plasma membrane with Imaris software. Error bars are given as +s.e. of ten independent samples each. *P<0.05, ****P<0.001.

We found that transiently expressed bio-active fluorescence-tagged zebrafish Wnt8 formed stable clusters at the cytoplasmic membrane of gastrula-stage embryos (Fig. 2A), thereby inducing the formation of aggregates of the co-receptor Lrp6 (Fig. 2B,E and supplementary material Fig. S3A-C) and its kinase CK1γ (Fig. 2E,F and supplementary material Fig. S3D-F), as well as members of the transducer complex such as GSK3β (Fig. 2D,G and supplementary material Fig. S3G-I), β-Catenin (Fig. 2H,K and supplementary material Fig. S3J-L), Axin1 (Fig. 2I,K and supplementary material Fig. S3M-O) and Dvl2 (Fig. 2J,M and supplementary material Fig. S3P-R), suggesting the formation of an active signalosome at the cytoplasmic membrane in zebrafish (for controls see supplementary material Fig. S4).

Dvl2 was recruited less efficiently to Wnt8 signalosomes (Fig. 2G). We reasoned that the endogenous concentration of Fz might be limiting because this Fz is the direct binding partner of Dvl in this complex. Expression of Fz1 in zebrafish embryos, indeed, led to an efficient recruitment of Dvl2 to the plasma membrane (Fig. 2H,I), and co-expression of Wnt8 and Fz1 induced the formation of Dvl2 clusters at the cell surface (Fig. 2J supplementary material Movie 1), which is comparable to the Wnt induced signalosomes described earlier (Fig. 2A-G). To our surprise, expression of Ap2μ2 and Fz1 induced analogous clustering of Dvl2 at the plasma membrane independently of Wnt (Fig. 2K). Consistently, in Ap2μ2 double-morphant embryos, size and number of Wnt8- and Dvl2-positive signalosomes were significantly reduced, although Fz1 was co-expressed (Fig. 2L,M). We found only one-third of induced signalosomes in Ap2μ2 morphant embryos (Fig. 2N) with the average diameter of the signalosome reduced twofold (Fig. 2O) compared to control embryos, suggesting that Ap2μ2 is required and sufficient to generate and maintain Dvl2- and Fz1-dependent signalosomes at the plasma membrane.

### Wnt8 is internalized together with Dvl2, Lrp6 and β-Catenin before intracellular separation occurs

**Fig. 3.**
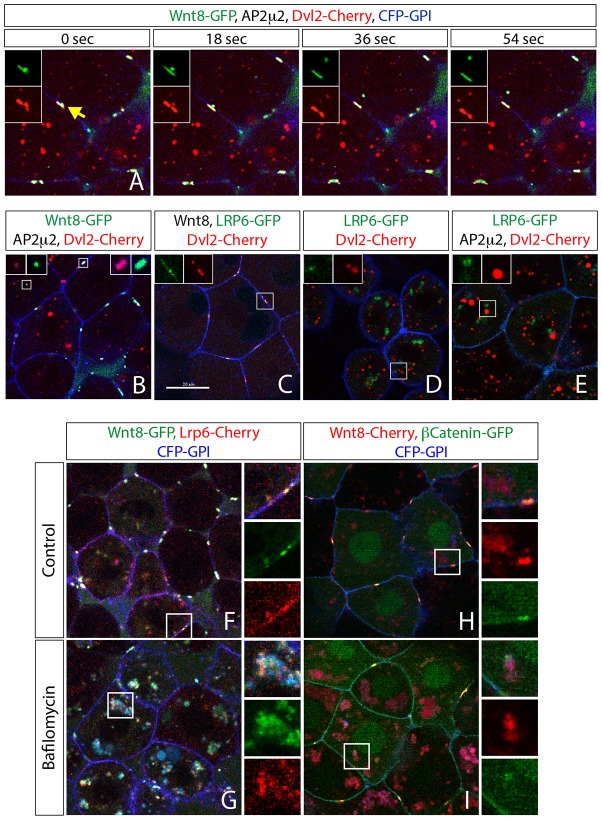
**Cell autonomous endocytosis of the signalosome.** Confocal microscopy analysis of live zebrafish embryos that express 2 ng mRNA of the indicated constructs at 30–50% epiboly stage; all constructs were used at 2 ng, except Wnt8 (1 ng), CFP-GPI (1,2 ng) and Ap2μ2 (1,6 ng). Confocal images represent single *z*-sections. Constructs were expressed as indicated and are shown in the indicated colors together with membrane markers CFP-GPI (blue). (A) Still pictures of a time-lapse analysis (from supplementary material Movie 2) of endocytosis of Wnt8 (green) together with Dvl2 (red) from a signalosome. (B–E) Dvl2 does neither colocalize with Wnt8 nor with Lrp6, unless in the plasma membrane after induction by Wnt8 (B,C). (F–I) Embryos treated with 150 nM bafilomycin A1 for 1 hour before scanning show colocalization of Wnt8 with Lrp6 (F,G) or β-Catenin (H,I). Arrow in A indicates a membranous cluster of Wnt8 and Dvl2 that is going to be internalized. Magnified images represent boxed areas with split channels related to the indicated color code for injected constructs.

In addition to the clusters of Dvl2 and Fz1 at the plasma membrane induced by either Wnt8 or Ap2μ2 (Fig. 2J,K yellow arrows), imaging analysis revealed intracellular Dvl2 punctae (Fig. 2J-L red arrows), which hardly ever colocalized with Wnt8 (Fig. 2L green arrow). We reasoned that these intracellular aggregates originate from a Dvl2 pool that was previously recruited to the membrane and, therefore, must have undergone an internalization event. To test the hypothesis that intracellular clusters form after signalosome endocytosis, we performed high-speed time-lapse imaging analysis of the dynamic signalosomes stimulated by Wnt8 and Ap2μ2 in zebrafish embryos. Indeed, we could monitor the internalization of Dvl2 together with Wnt8 every 25.5 minutes per cell, which equates to 0.04 endocytic events per cell and minute (Fig. 3A and supplementary material Movie 2; *n* = 65 events in 165 cells monitored). However, we found that only fragments of the signalosome were internalized, whereas the main part remained attached to the membrane. This suggests a flow-type process in which parts of the signalosome are endocytosed continuously and, in parallel, the signalosome becomes recharged by recruitment of new intracellular Dvl2. Noticeable, in all images analyzed, we barely detected intracellular colocalization of Dvl2 aggregates with Wnt8 or Lrp6 after the fast endocytosis of both proteins (Fig. 3B-E), suggesting either a quick separation and/or degradation step of the ligand-receptor complex after endocytosis or a process beyond detection limit.

Therefore, we decreased endosomal acidification by treating embryos with bafilomycin A1 to slow down endosomal maturation into late endosomes (Hurtado-Lorenzo et al., 2006) and to block potential degradation of Wnt8 in lysosomes. Indeed, upon bafilomycin treatment we found accumulation of Wnt8 in the cytoplasm (Fig. 3G,I and supplementary material Fig. S3D,E). Similarly, the co-receptor Lrp6 was enriched in the same cytoplasmic structures (Fig. 3F,G). In addition, we found β-Catenin colocalizes with Wnt8 at the plasma membrane but accumulates juxtaposed to Wnt8-positive cytoplasmic structures after treatment with bafilomycin (Fig. 3H,I). This suggests that, upon Wnt stimulation, parts of the signalosome – including the ligand-receptor complex and the transducer complex linked to Dvl2 – undergo rapid endocytosis that depends on Ap2 function. Most of these events are probably very fast and the fluorescent signal of endocytic events may be below the detection limit of regular laser-scanning microscopy. However, we can reliably visualize internalization when the endocytic route is chemically blocked and the signalosome components are subsequently accumulating in enlarged endosomes.

### Wnt8 is internalized together with Dvl2 and Lrp6 in a paracrine fashion that depends on Ap2 function

**Fig. 4.**
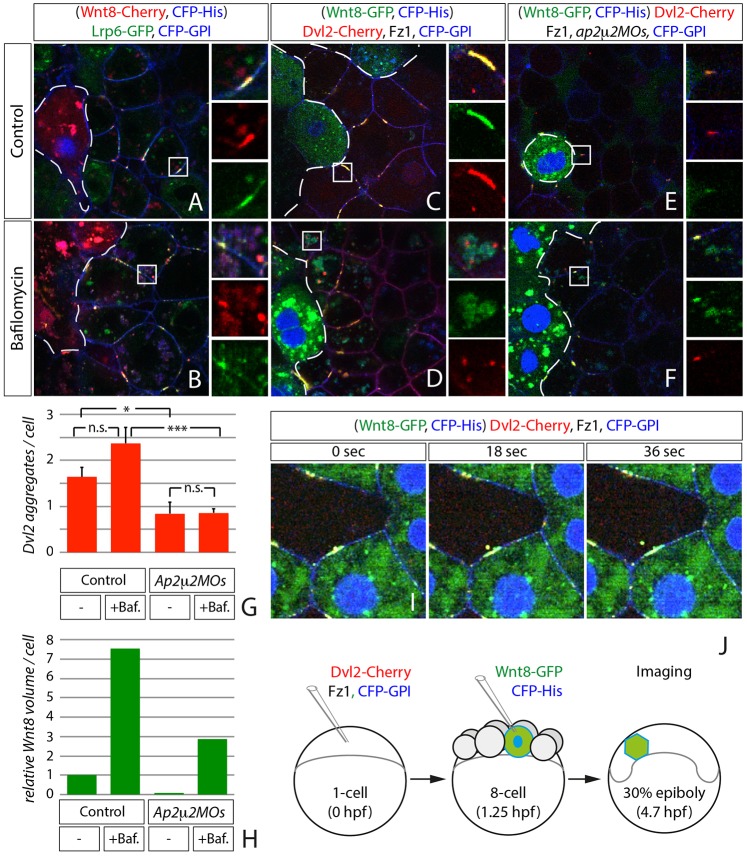
**Clonal analysis of signalosome internalization.** Confocal microscopy analysis of live zebrafish embryos injected with a set of mRNAs of indicated constructs (2 ng Dvl2, 1,2 ng of CFP-GPI, 1,6 ng Ap2μ2 or Fz1) at one-cell stage and later at eight-cell stage injected with 0.25 ng Wnt8 and 0.1 ng CFP-Histone H2B into one cell only. For convenience, Wnt8-producing clones were marked by nuclear CFP (indicated by dashed lines). Confocal images represent single *z*-sections. (A,C) Controls. (E) Co-injection of Ap2μ2 morpholinos (MOs) at one-cell stage. (B,D,F) Embryos were treated with bafilomycin A1 for 1 hour before scanning. Magnified images represent boxed areas with split channels related to the indicated color code for injected constructs. (G) Manual quantification of intracellular Dvl2-Cherry vesicles in cells next to Wnt8 clone from (C–F). Error bars are given as +s.e. of ten independent samples each. *P<0.05; *** *P*<0.005; n.s., not significant (*P*≥0.05). (H) Automatic quantification after surface application (using Imaris software) of intracellular Wnt8-GFP total volume in 15 responding cells of three different embryos. Represented in volume per cell normalized to volume in control cells (1 = 25,6 µm^3^). (I) Still pictures of a time-lapse analysis of endocytosis of Wnt8 (green) together with Dvl2 (red) from a signalosome (supplementary material Movie 3). (J) Experimental procedure to generate clones displayed in (A–F and (I).

Since it had been suggested that Wnt ligands are endocytosed in an autocrine fashion (Port and Basler, 2010) in order to be secreted on exosomes (Gross et al., 2012), we aimed to distinguish between autocrine and paracrine internalization of Wnt8. Therefore, we generated small ligand-secreting cell clones by overexpression of Wnt8 in one out of eight blastomers in a Lrp6-positive and a Dvl2- and Fz1-positive zebrafish embryo (Fig. 4J). In cells adjacent to a Wnt-producing clone we found clusters positive for Wnt8 and Lrp6 or for Dvl2 at the plasma membrane (Fig. 4A,C and supplementary material Fig. S5A), suggesting the paracrine induction of signalosomes by Wnt8. Quantification of internalization events of Wnt8 together with Dvl2 revealed one endocytic event per receiving cell every 33 minutes (Fig. 4I,J and supplementary material Movie 3; *n* = 30 cells). After treatment with bafilomycin A1, we observed overlapping intracellular accumulation of Wnt8 and Lrp6 compared to control embryos (Fig. 4A,B and supplementary material Fig. S5B), whereas Dvl2 punctae rather appeared juxtaposed to Wnt8 aggregates (Fig. 4C, D). We quantified the number of endocytosed Dvl2 punctae in cells next to the Wnt8 source and found them increased 1.7-fold after acidification blockage (Fig. 4G), also suggesting that a fraction of the Dvl2 population escapes our detection under control conditions. Consistently, we found that signalosome formation was reduced in Ap2μ2 morphant embryos (Fig. 4E). Additionally, we found that Dvl2 was only half as much internalized after knockdown of Ap2μ2 function in receiving cells independent of the bafilomycin treatment (Fig. 4E-G). The total volume of internalized Wnt8 became almost undetectable in Ap2μ2 morphant embryos compared to control (Fig. 4A,C,E,H). In bafilomycin-treated embryos, however, the intracellular volume of Wnt8 aggregates increased sixfold compared to control-treated embryos (Fig. 4B,D,H) and Wnt8 accumulation was again reduced threefold in Ap2μ2 morphant embryos after bafilomycin treatment but still detectable (Fig. 4F,H). The remaining intracellular Wnt8 might be a result of the partial knockdown of Ap2μ2 function using the morpholino approach. These results suggest that Wnt8 and Dvl2 internalization requires Ap2 function and points to an essential role of endocytosis during Wnt/β-Catenin signaling.

### Wnt8 colocalizes with Rab7-positive late endosomes

**Fig. 5.**
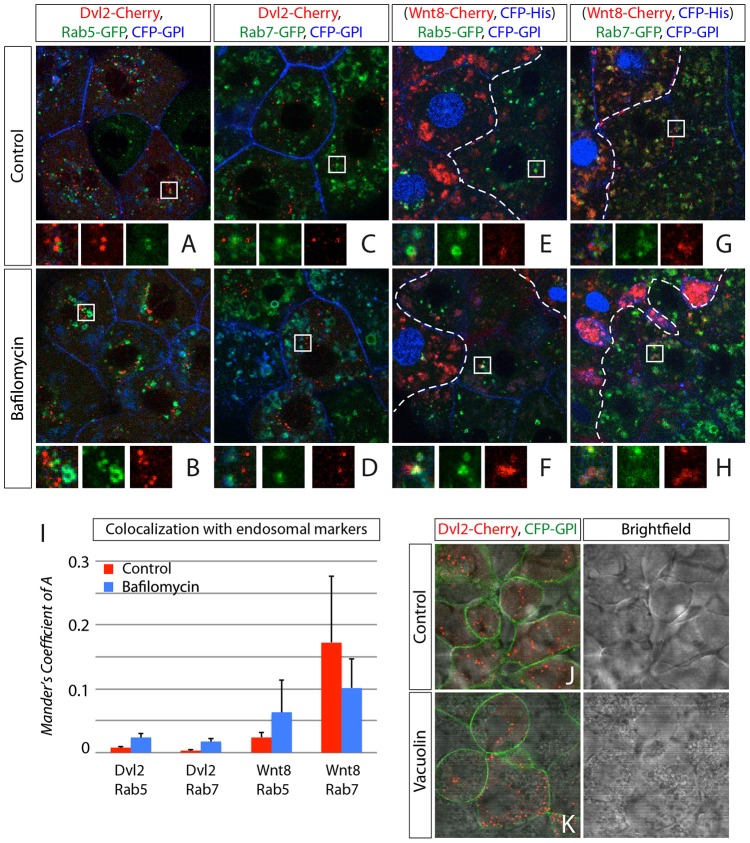
**Colocalization of Wnt8 and Dvl2 with endosomal markers.** Confocal microscopy analysis of Rab5-GFP or Rab7-GFP transgenic zebrafish embryos that express mRNA of indicated constructs at 30–50% epiboly stages. (A–D) Expression of Dvl2 in Rab5-GFP (A,B) or Rab7-GFP (C,D) zebrafish transgenic line. Dashed lines in E–H indicate the border between Wnt8-positive cell clone (left) and host tissue (right). Confocal images represent single *z*-sections. Constructs were expressed as indicated and are shown in the indicated colors together with membrane marker CFP-GPI (blue). (E–H) Clonal expression of Wnt8 together with nuclear marker His-CFP in Rab5-GFP (A,B) or Rab7-GFP (C,D) zebrafish transgenic line transiently expressing CFP-GPI. (I) Quantification of colocalization of Dvl2 with Rab5 and Wnt8 with Rab7. Analysis was performed using the Imaris Coloc software module function. Thresholds were set manually and Manders Coefficient for either Dvl2 or Wnt8 was calculated automatically. Error bars represent +s.e. of five independent samples each. (J) Control embryos treated with 0.3% DMSO, (K) Embryos treated with Vacuolin-1 in 0.3% DMSO for 1.5 hours before scanning.

To investigate the intracellular localization of the transducer complex and the ligand-receptor complex, we made use of stable transgenic zebrafish lines expressing fluorescently tagged Rab5 or Rab7 (Clark et al., 2011). We quantified the colocalization of endosomal markers with either Dvl2 or Wnt8 by measuring the Manders colocalization coefficient (Coefficient of 1 = full colocalization, 0 = random localization). We found almost no colocalization of Dvl2 with either Rab5 or Rab7 signals shown by a Manders coefficient of 0.008±0.001 or 0.003±0.002 (mean±s.e.m), respectively (Fig. 5A,C,I). A minimal increase of the colocalization coefficient was observed in both cases after treatment with bafilomycin (Rab5: 0.023±0.006; Rab7: 0.017±0.005 (Fig. 5B,D,I), suggesting a separation of Dvl2-positive aggregates from early and late endosomes.

The total pool of the analyzed Wnt8 showed some but little colocalization with Rab5 (0.024±0.008) and strong overlap with Rab7 (0.172±0.041; Fig. 5E,G,I). This distribution shifted towards a higher correlation between Wnt8 aggregates and Rab5 positive vesicles after bafilomycin treatment (Rab5: 0.063±0.005, Rab7: 0.104±0.046; Fig. 5F,H,I), suggesting that Wnt8 colocalize with early and late endosomes. To provide further evidence that the formation of Dvl2 aggregates is independent of endosomes, we treated embryos with the small molecule inhibitor Vacuolin-1, leading to a homotypic fusion of endosomes and lysosomes (Cerny et al., 2004). Although endosomes, including multi-vesicular bodies, fuse to gigantic vesicles in gastrula cells, the appearance of Dvl2 aggregates is not altered and Dvl2 aggregates do not overlap with any of the visible vacuoles, whereas Wnt8 was not detectable in the receiving cells after the treatment (Fig. 5J,K and data not shown). These results support our observation that the entire Lrp6-signalosome becomes internalized. Subsequently, the intracellular part of the signalosome, the Dvl-positive transducer complex, dissociates from the Wnt8-positive ligand-receptor complex to form intracellular aggregates, while the ligand-receptor complex is routed to Rab7-positive late endosomes for degradation or recycling. The little colocalization of Dvl2- and Rab5-positive vesicles − even in bafilomycin-treated embryos − suggests that dissociation of the intracellular transducer complex from the ligand-receptor complex occurs at a very early time point after endocytosis during zebrafish gastrulation.

### Ap2μ2-mediated endocytosis is required for Dvl2 stability

**Fig. 6.**
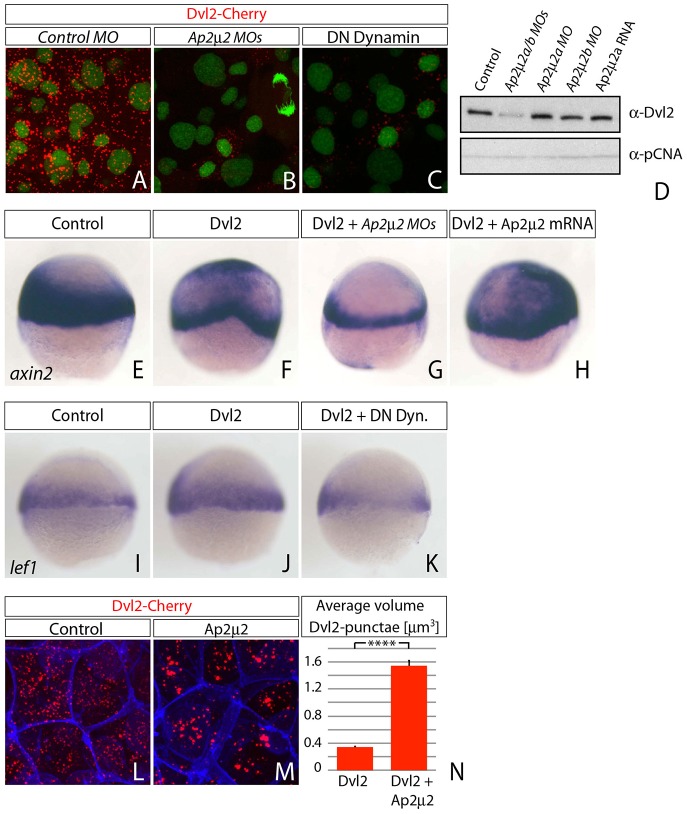
**Dvl2 stability and activity depends on Ap2μ2 function.** (A–C,L,M) Confocal microscopy analysis of live zebrafish embryos that express synthetic mRNA of the indicated constructs and morpholino (MO) oligomers against *ap2μ2a/b* at 40% epiboly stage. (A–C) Intracellular clusters of Dvl2 are hardly detectable in Ap2μ2 double-morphant embryos (B) and in embryos that express dominant-negative (DN) Dyn (C) compared to control. (D) Western blot analysis of Ap2m2 morphant embryos and Ap2m2-mRNA-expressing embryos shows a reduction of endogenous Dvl2 in double-morphant embryos. PCNA was used as loading control. (E–K) *In situ* hybridization with *axin2* and *lef1* probed in embryos injected with indicated constructs at blastula-stage. Embryos shown are representative of 30–40 stained embryos; the lateral view is shown (the dorsal side is on the right). (L,M) Ectopic expression of Ap2μ2 mRNA leads to increased size of Dvl2 aggregate. (N) Quantification of data shown in L and M. Error bars are given as +s.e. of five independent samples each. ****P<0.001.

In the past, Dvl has been reported to dynamically polymerize through its DIX domain to form cytoplasmic punctae – an essential process during Wnt/β-Catenin signaling in cell culture and in *Drosophila melanogaster* (Axelrod et al., 1998; Schwarz-Romond et al., 2007a; Schwarz-Romond et al., 2007b; Smalley et al., 1999). However, it is still unclear what the origin and the function of these punctae are. We reasoned that these aggregates might be assemblies of the transducer complex still linked to Dvl2 after release from the Wnt/Lrp-positive endosomes. Therefore, we tested whether endocytosis is required for Dvl2 aggregate formation. In Ap2μ2 morphant embryos, we observed a decreased amount of cytosolic Dvl2 aggregates compared to control embryos (Fig. 6A,B), similar to embryos impaired for endocytosis by overexpression of the dominant-negative Dynamin2-K44A mutant (DN Dynamin; Fig. 6C). Consistently, we detected less endogenous Dvl2 protein by immunoblot analysis of Ap2μ2 knockdown embryos (Fig. 6D), pointing to a decreased stability of Dvl2 protein due to impaired endocytosis. Furthermore, expression of target genes was ectopically induced in Dvl2-injected embryos (Fig. 6E,F and I,J) and reduced in embryos co-injected with either Ap2μ2 morpholinos or dominant-negative Dynamin (Fig. 6G,K). However, overexpression of Ap2μ2 increased Dvl2 cluster size threefold (Fig. 6L-N) and led to the mutual activation of *axin2* expression (Fig. 6H). Notably, Dvl2 protein stability was unaltered after Ap2μ2 overexpression (Fig. 6D). Together, these results show that Dvl2 stability and, therefore, Wnt/β-Catenin signaling depend on Ap2μ2 mediated endocytosis in zebrafish.

### Binding of Ap2μ2 to Dvl2 is required for Wnt/β-Catenin activity

**Fig. 7.**
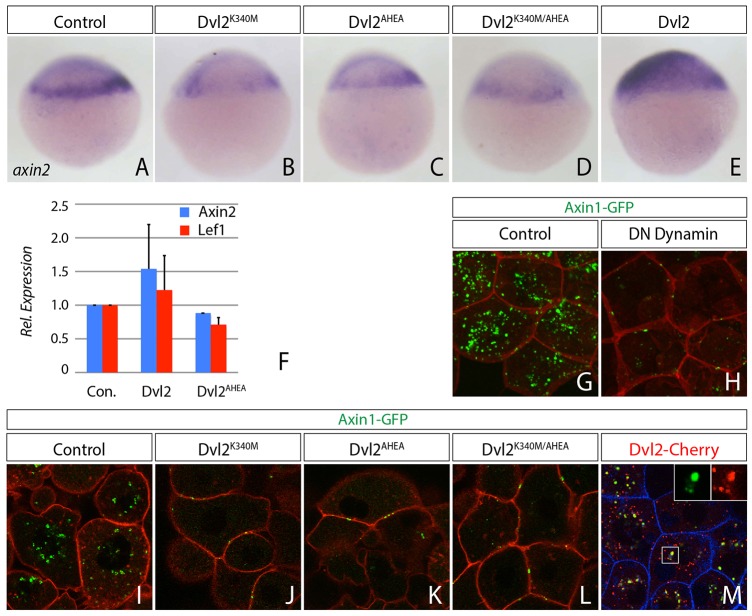
**Dvl2 internalization and activity is dependent on Ap2μ2-binding sites.** (A–E) *In situ* hybridization shows decreased activity of the Wnt/β-catenin signaling pathway reflected by the induction of *axin2* expression in blastula-stage embryos at 30% epiboly. Embryos were injected with indicated constructs; the lateral view is shown (the dorsal side is on the right). (A) *n* = 72; (B) 33/39; (C) *n* = 27/29; (D) *n* = 23/29; (E) *n* = 33/35. (F) Relative target gene expression analyzed by real-time PCR with primers against Axin2 (blue) and Lef1 (red) on embryonic cDNA injected with the indicated constructs. Error bars are given as +s.e. of two independent experiments scanned in duplicates. (G,H) Blockage of Dynamin-dependent endocytosis reduces the Axin1-GFP signal in the cytoplasm. Confocal images show 12 µm *z***-**stacks of live embryos at 40% epiboly stage. Indicated constructs were injected at 1 ng as synthetic mRNA, except Axin1 (2 ng), at one-cell stage. (I–M) Axin1 is blocked at the cytoplasmic membrane and its stability reduced when interaction between Dvl2 and AP2μ2 is impaired. (I) Control. Axin1 aggregates in the cytoplasm. (J–L) Co-expression of Dvl2 single- (J,K) and double-mutants (L) together with Axin1. (M) Colocalization of Axin1 with wild-type Dvl2 in cytoplasmic punctae. Confocal images represent single *z*-sections of live embryos at 40% epiboly stage. Indicated constructs were injected as synthetic mRNA (2 ng each) at one-cell stage.

To test whether signaling and internalization of the transducer complex requires Ap2μ2 binding to Dvl2 in zebrafish, we introduced two point mutations into Dvl2, one in the DEP domain (K340M) and another in the tyrosine-based YxxΦ sorting motive (AHEA) at the C-terminus, because both domains were described to be essential binding sites for Ap2μ2 in cell culture (Yu et al., 2007; Yu et al., 2010). We found that expression of *axin2* and *lef1* was reduced when Dvl2 single-mutants and the double mutant were co-expressed (Fig. 7A-E,F). This suggests that interaction of Dvl2 and Ap2μ2 is necessary for the activation of Wnt/β-Catenin signaling. Next, we asked whether the inability of Dvl2 mutants to activate Wnt/β-Catenin signaling is linked to impaired endocytosis. Therefore, we co-expressed Axin1 with the Dvl2 mutants and analyzed its subcellular behavior. We found that co-expression of the Dvl2 mutants led to a reduction of Axin1-GFP fluorescence and the remaining Axin1-GFP-formed aggregates at the cytoplasmic membrane (Fig. 7I-L). Notably, in the Dvl2 mutants the domain that interacts with Axin1 (DIX-domain) is intact. However, we found that wild-type Dvl2 colocalized with Axin1 in cytoplasmic punctae (Fig. 7M). Next we addressed whether blockage of endocytosis impairs Axin1 localization. We found that co-expression of DN Dynamin reduced Axin1-GFP fluorescence and Axin1 was found predominantly at the plasma membrane (Fig. 7G,H), similarly to our observation with the Dvl2 mutants. These findings suggest that the interaction of Dvl2 with Ap2μ2 is necessary to allow endocytosis of the transducer complex, which is a prerequisite for Wnt/β-Catenin signaling.

### Members of the transducer complex cluster intracellularly with Dvl2

**Fig. 8.**
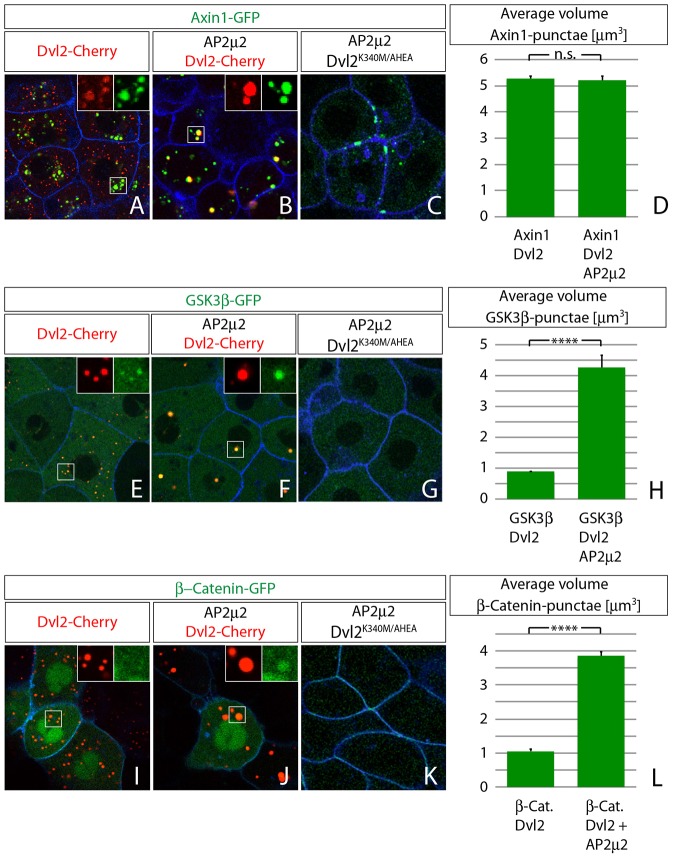
**Members of the transducer complex cluster intracellularly with Dvl2.** Confocal Images represent single *z*-sections of live embryos at 40% epiboly stage. Indicated constructs were injected as synthetic mRNA (2 ng each, 1,6 ng of Ap2μ2, 1,2 ng CFP-GPI) at one-cell stage and displayed with indicated colors. Subcellular distribution of Axin1 (A–D), GSK3β (D–H) and β-catenin (I–L) in the context of Dvl2 co-expression. (A,B) Axin1 is distributed as intracellular punctae that colocalize with Dvl2 (A) even in the presence of Ap2μ2 (B). (C) Axin1 is found predominantly in plasma membrane clusters after co-expression of Ap2μ2 and Dvl^K340M/AHEA^ double mutant mRNA. (D) Automatic quantification of punctae size in (A,B) as volume after surface application using Imaris software. GSK3β (D–H) clusters in Dvl2-positive aggregates and colocalization can be improved by co-expression of Ap2μ2 mRNA. (G) GSK3β distribution is unaltered (compare with supplementary material Fig. S3E) after co-expression of Ap2μ2 and Dvl^K340M/AHEA^ double mutant mRNA. (H) Quantification of punctae size in (E,F). (I) β-Catenin clusters are less apparent in Dvl2-positive aggregates, but colocalization can be improved by co-expression of Ap2μ2 mRNA (J). (G) β-Catenin distribution is unaltered (compare with supplementary material Fig. S3G) after co-expression of Ap2μ2 and Dvl^K340M/AHEA^ double mutant mRNA. (L) Quantification of punctae size in (E,F). Error bars are given as +s.e. of ten independent samples each. ****P<0.001; n.s., not significant (*P*≥0.05).

Next we asked whether these Dvl2 aggregates gain their Wnt/β-Catenin signaling activity by holding other members of the transducer complex together. Therefore, fluorescently tagged versions of Axin1, GSK3β and β-Catenin were imaged in live zebrafish embryos to investigate their dynamics and interaction with Dvl2 *in vivo*. The scaffold protein Axin1 was described to bind directly to the DIX domain of Dvl2 (Schwarz-Romond et al., 2007a) and, indeed, co-expression with Dvl2 showed colocalization of the two proteins in clusters independently of co-expression of Ap2μ2 (Fig. 8A-D; supplementary material Fig. S3A-C). We were also interested whether Ap2μ2 is able to compensate for the inhibited endocytosis of Axin1 after co-expression with the Dvl2^K340M/AHEA^ double-mutant (Fig. 7L). However, Axin1 localized predominantly to the plasma membrane when expressed together with Ap2μ2 and Dvl2^K340M/AHEA^ (Fig. 8C). GSK3β, the main Ser/Thr-kinase for β-Catenin, appeared evenly distributed in the cytoplasm when expressed in embryos (supplementary material Fig. S4E). However, Dvl2 recruited GSK3β into punctae upon overexpression (Fig. 8E), and co-expression of Ap2μ2 formed four times larger clusters of Dvl2 and GSK3β (Fig. 8F,H) whereas the change of wild-type Dvl2 to Dvl2^K340M/AHEA^ did not lead to any cytoplasmic clustering of GSK3β (Fig. 8G). β-Catenin – as the main effector of canonical Wnt/β-Catenin signaling and as part of adherens junctions – did localize to the cytoplasmic membrane (supplementary material Fig. S4G). Expression of Dvl2 did not lead to obvious colocalization with β-Catenin (Fig. 8I). However, when Dvl2 and Ap2μ2 were co-expressed, β-Catenin was efficiently recruited to the same clusters, which increased in size four times compared to Dvl2 clusters alone (Fig. 8J,L); the co-expression of Ap2μ2 and Dvl2^K340M/AHEA^ did not lead to any cytoplasmic clustering of β-Catenin (Fig. 8K).

These results suggest that Dvl2-positive clusters represent an intracellular transducer complex that includes Axin1, GSK3β and β-catenin and that is endocytosed. We hypothesize that the transducer complex resembles a pre-stage of the β-Catenin destruction complexes in its inactive form that allows prolonged maintenance of Wnt/β-Catenin signaling following signalosome endocytosis.

## DISCUSSION

### Ap2μ2 function during Lrp6 signalsome formation

In this study, we investigated the cell biological mechanism of Wnt/β–catenin signaling by using high-resolution confocal imaging in zebrafish. The aim of our study was to investigate the process of Lrp6-signalosome formation and the subsequent endocytosis of this Lrp6-signalosome with regard to Wnt/β-Catenin signaling on a subcellular level but in a living vertebrate organism. So far, signalosomes of canonical Wnt signaling have been reported only in tissue culture cells (Bilic et al., 2007; Kim et al., 2013a). In zebrafish embryos at blastula stage, we found membranous aggregates of ligands, receptors and transducers of the Wnt/β-Catenin pathway in Wnt-responding cells and, in accordance with previous studies (Bilic et al., 2007), termed these structures Lrp6-signalosomes. We were able to induce the formation of Lrp6-signalosomes and to enhance Wnt/β-Catenin signaling independently of the ligand by overexpressing Ap2μ2, which implies that Ap2 and endocytosis are crucial during Wnt/β-Catenin signaling. Ap2 had been suggested to play a role in various other signaling pathways, such as the Fgf and the Tgf-β signaling pathways in zebrafish embryos (Umasankar et al., 2012). Thus, we hypothesize that Ap2μ2 is a central hub protein that connects two important cell biological processes: the regulation of transduction cascades and the process of endocytosis.

### Endocytosis and signaling

We found that knockdown of Ap2μ2 leads to a reduction of Lrp6-signalosome formation and endocytosis, which leads to a decrease of Wnt/β-Catenin signaling. Previous studies in *Drosophila* and in mouse and human cell lines are controversial in their conclusions concerning the type of endocytosis to be required for Wnt/β-Catenin signaling and whether endocytosis is necessary for signaling. (Blitzer and Nusse, 2006; Glinka et al., 2011; Jiang et al., 2012; Piddini et al., 2005; Yamamoto et al., 2006; Yamamoto et al., 2008a). Initially, we had detected very little intracellular signal of fluorescently tagged Wnt8 in the receiving cells of our clonal imaging assay. However, by blocking the maturation of endocytic vesicles with bafilomycin A1, large aggregates of Wnt colocalizing with Lrp6 became visible, suggesting a fast and transient event of endocytosis that had escaped previous analysis. It had been proposed that clathrin-coated vesicles are able to border only one to three ligand-bound receptors (Cocucci et al., 2012). We were able to monitor these processes by using high-resolution confocal microscopy in combination with zebrafish embryos, which have completely translucent and large cells (with a diameter of 15–20 µm) during blastula stages. However, a number of endocytic events might have escaped our detection.

When we blocked endocytosis at the plasma membrane by knocking down the function of Ap2μ2 or by expressing dominant-negative Dynamin2, we did not find any comparable intracellular or extracellular accumulation of Wnt8. At the same time, we found the formation of Lrp6-signalosomes reduced. This result was confirmed by a recent study using mammalian cell lines, which describes interaction of Ap2μ2 with the Lrp6 co-receptor (Kim et al., 2013a). The authors of this study suggest that, after Wnt activation, clathrin accumulates at the site of Lrp6 assembly and Ap2 function is essential for this aggregate formation but depends on the presence of phosphatidylinositol (4,5)-bisphosphate. In contrast to our data, internalization of Lrp6-signalosomes in response to Wnt signal activation was not observed.

### The μ2 subunit is required to protect Dvl2 from degradation

In this study, we focused on the previously shown interaction of Ap2μ2 with Dvl2 (Yu et al., 2007; Yu et al., 2010), which has dramatic effects on Dvl2 stability and function during Wnt/β-Catenin signaling. Our data show that formation and function of Dvl2 aggregates depends on the interaction with Ap2μ2, suggesting that cytoplasmic Dvl2 aggregates all stem from a previous internalization event. Taken that high concentrations of Dvl2 can activate Wnt/β-Catenin target genes, we presume that interference with binding between Dvl2 and Ap2μ2 blocks endocytosis of the signal transducer complex, and that Dvl2 is subsequently degraded. Indeed, previous experiments using the mouse neuronal cell line SN4741 in culture with hyperosmolaric sucrose have suggested that endocytosis is important to maintain the Dvl pool (Bryja et al., 2007). So, why does endocytosis promote Dvl2 stability? It has been suggsted that Dvl2 is quickly degraded and needs stabilizing binding partners (Gao et al., 2010). At the plasma membrane Dvl2 binds to Ap2μ2, which stabilizes Dvl2 protein (Fig. 6). After endocytosis, cytoplasmic Dvl2 aggregates form the transducer complexes, a combination of components of the inactive β-Catenin destruction complex and Dvl2. This is supported by our data showing colocalization of Dvl2 with other members of the transducer complex such as Axin, GSK3β and β-Catenin in the cytoplasm, which has been reported in human cell culture before (Metcalfe et al., 2010). The transducer complex might be important to protect Dvl2 from degradation after removal from the membrane. Indeed the stability of Dvl is controlled by proteasomal and lysosomal degradation and Dvl–Axin polymer formation prevents its constitutive proteolytic destruction (Cliffe et al., 2003). Furthermore, a recent report suggested that activation of the Wnt pathway leads to stabilization of Dvl by de-ubiquitylation in the human cell line HEK293T (Jung et al., 2013). We, therefore, hypothesize that Ap2μ2-mediated recruitment of Dvl2 to Lrp6-signalosomes and the subsequent formation of the transducer complex stabilize Dvl2 and, thus, promote Wnt/β-Catenin signaling in zebrafish.

### Endocytic routing of Wnt8 and Dvl2 after internalization

Studies in HeLa cells suggested that the entire Lrp6 signalosome localizes to MVBs or lysosomes by showing colocalization of Dvl with Axin1, GSK3β and Lysotracker (Taelman et al., 2010; Vinyoles et al., 2014). Indeed, we demonstrate that Wnt8 together with Lrp6 is taken up by endocytosis and can be found in early endosomes and subsequently in late endosomes. However, our data show that Dvl takes a different route: Dvl2 aggregates do not overlap with Rab5 or Rab7 and do not alter their shape in combination with the small chemical Vacuolin-1 (Cerny et al., 2004), this suggests that after endocytosis, intracellular signalosome components dissociate from the ligand-receptor loaded vesicles but temporarily stay polymerized in the cytoplasm. We speculate that the dismantling of the Clathrin coat including Ap2 with its subunit μ2 might be linked to the dissociation of the Dvl2-positive signalosome from the vesicle. After endocytosis, Clathrin-coated vesicles are dismantled by the activity of the heatshock cognate protein Hsc70 (Kirchhausen, 2002). In this process Ap2 and Clathrin are translocated back to the plasma membrane. Our data indicate that Dvl2 exists as a member of the intracellular transducer complex. This hypothesis is in accordance with previous studies recognizing the biological relevance of intracellular Dvl2 aggregates – the so-called Dvl2 punctae (Schwarz-Romond et al., 2007b; Smalley et al., 1999). In addition, it was recently proposed that the destruction complex is a very stable structure and is maintained after assembly in the cytoplasm (Li et al., 2012). Furthermore, this destruction complex might be converted from an active state in which β-Catenin is routed for degradation into an inactive state by PP1-mediated dephosphorylation of the scaffold protein Axin1 (Kim et al., 2013b).

**Fig. 9.**
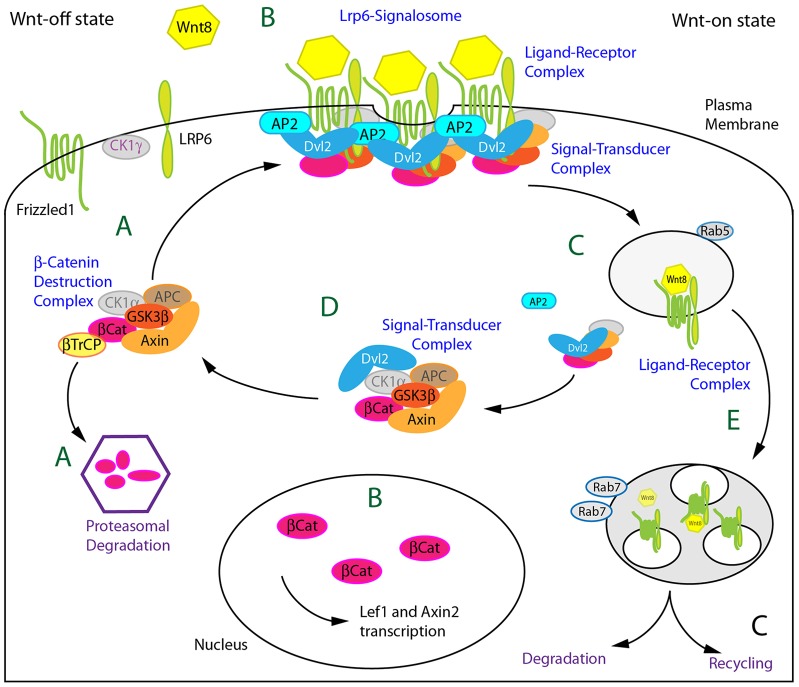
**Model for Ap2μ2 function.** Schematic drawing of Ap2μ2 action during canonical Wnt/β-Catenin-signaling. (A) In the Wnt-off state, the β-Catenin destruction complex is active and degrades β-Catenin. (B) In the Wnt-on situation, ligand is bound to Fz receptor and Lrp6 co-receptor, thereby inducing Ap2-dependent recruitment of Dvl2 to Fz and signalosome formation at the cytoplasmic membrane. (C) Subsequent internalization is mediated by the interaction of Dvl2 with Ap2μ2 and leads to the sequestration of Axin, CK1γ, GSK3β and β-Catenin in cytoplasmic aggregates. The ligand-receptor complex is routed to late endosomes for degradation or recycling (E), whereas the signal-transducer complex (D) maintains signaling activity by stabilizing Dvl2 and by, for example, keeping GSK3β inactive. Subsequently, the destruction complex becomes activated again by, for example, phosphorylation of Axin1, leading to the initial Wnt-off state (A).

In summary, we propose a model (Fig. 9), in which an affinity balance exists between members of the β-Catenin destruction complex and Dvl2 between the cytoplasm and the plasma membrane. In the Wnt-off state (Fig. 9A) the balance is on the assembly of the destruction complex and the β-Catenin degradation. Binding of the Wnt ligand to receptors Fz1 and Lrp6 initiates clustering of the receptor complex (Fig. 9B). When the number of accumulated Fz1 receptors is high enough, Dvl2 is recruited to the receptor and binds to Ap2μ2. This may induce the polymerization and phosphorylation of Lrp6, which in turn initiates the recruitment of the Axin1-positive destruction complex – including GSK3β – to the receptor complex, leading to the formation of an activated signalosome (Bilic et al., 2007; Davidson et al., 2005). We propose that, subsequently, Dvl2 and Ap2μ2 together initiate endocytosis of fractions of the signalosome (Fig. 9C). The following Clathrin recycling process may be further required to split the intracellular Dvl2-positive signal transducer complex (the inactivated destruction complex) from the receptor complex, including Fz1, Lrp6 and the ligand. Ligand and receptors, subsequently, become either degraded in the lysosome or are recycled to the membrane (Fig. 9E). The signal transducer complex, however, dissociates from the early endosome (Fig. 9D), which leads to further maintenance of the Wnt-on state.

## Materials and Methods

### Zebrafish embryos and manipulation

All zebrafish husbandry and experimental procedures were performed in accordance with the German law on Animal Protection and were approved by Local Animal-Protection Committee (Regierungspräsidium Karlsruhe, Az.35-9185.64) and the Karlsruhe Institute of Technology (KIT). Breeding zebrafish (*Danio rerio*) were maintained at 28°C on a 14 h light/10 h dark cycle (Brand et al., 2002). To prevent pigment formation, embryos were raised in 0.2 mM 1-phenyl-2-thiourea (PTU, Sigma, St Louis, MO) after 20 hpf. The data we present in this study were acquired from analysis of KIT wild-type zebrafish AB2O2. Rab5-eGFP and Rab7-eGFP expressing transgenic lines were a kind gift from Brian Link.

Embryos were harvested and enzymatically dechorionated with Pronase (Sigma-Aldrich) at one-cell stage before injection. All indicated constructs were injected at one-cell stage or, for clone formation, into one of 8–16 cells as capped mRNA, *in-vitro* transcribed with the mMessage Machine Kit (Ambion).

### Constructs and morpholinos

zAp2μ2a/b morpholino oligomers (Ap2μ2a: 5′-CCAATCATGGTGGCTGGTCCTCAAA; Ap2μ2b: 5′-CCTCCAATCATGGTGGCGGTTAAAC) spanning the ATG were obtained from GeneTools LTD.

All expression constructs were subcloned in pCS2+ (Rupp et al., 1994). zDvl2 (Waxman et al., 2004); # 17086, xGSK3β-GFP (Taelman et al., 2010); #29680, xβ-Catenin-GFP (Miller and Moon, 1997); #16839, zWnt8 ORF1 (Lekven et al., 2001); #17048 are from Addgene. hAxin1-GFP, hCK1γ-GFP, hLrp6-GFP and hLrp6-Cherry were kindly provided from Gary Davidson. zWnt8-GFP was kindly provided by Michael Brand (Rhinn et al., 2005). CFP-GPI, His-CFP (HistoneH2B-CFP), xRab5-GFP were kind gifts from Jim Smith (Hagemann et al., 2009).

Ap2μ2a was amplified from 24 hpf zebrafish embryo cDNA library with primer A (5′-CCATGGAATGTGAGGTTTGA-3′) and B (5′-GAGTATGACCGATGGGGAAA-3′) using Pfu polymerase (Promega) and the resulting product was introduced into pCS2+ for Ap2μ2a or into pGlyserteC-TFP [kind gift from Uwe Strähle (Roostalu and Strähle, 2012)] for Ap2μ2a-TFP.

For zDvl2-Cherry and zDvl2 mutants, all PCR reactions were performed per manufacturer directions using Phusion High Fidelity polymerase (New England Biolabs). Restriction sites were introduced to the N- and C-termini of zDvl2 (plasmid from Thermo Scientific) (*Hin*dIII and *Kpn*I, respectively; restriction sites are shown as lowercase letters) by amplification with primer A (5′-GGCaagcttGCACCATGGCGGAGACCAAGATAATTTATC-3′) and B (5′-CGGggtaccTTACATCACATCCACAAAAAACTCACTGg-3) and the resulting product introduced into pmCherry C-1 (Clontech). Mutagenesis to introduce the K340M mutation was performed in two steps. First a left flanking fragment was generated using primer A and primer C (5′-CATTGGGAATGGTGATCATCAGCCACATTCGATCT-3′). The right flanking fragment was generated with primer B and D (5′-AGATCGAATGTGGCTGATGATCACCATTCCCAATG-3′). The resulting products were gel-purified and added at equal molar concentration to a second reaction containing primers A and B. The product of this fusion PCR was purified, introduced into pZero, and confirmed by sequencing to yield pZero-zDVL K340M. The Y55A and L554A mutations were introduced simultaneously in a similar two-step manner, using primers E (5′-GTAGCTGTAGTTAGAGGCCTCATGGGCTGGCGGAGGCTGGGTG-3′) and F (5′-CACCCAGCCTCCGCCAGCCCATGAGGCCTCTAACTACAGCTAC-3′) to produce pZero-zDVL AHEA.

The triple-mutant was generated by liberating a fragment from pZero-zDVL K340M by *Bam*HI digest and subsequent ligation into pZero-zDVL AHEA.

### Inhibitor treatment

Embryos were treated with 150 nM bafilomycin A1 (SAFSB1793-10UG, from VWR International) in 1% DMSO for 1 hour before scanning or with 30 µM Vacuolin-1 in 0.3% DMSO for 1.5 hours before scanning (V7139, from Sigma-Aldrich).

### *In situ* hybridization

Embryos were fixed in 4% paraformaldehyde in PBS. Whole-mount mRNA *in situ* hybridization was performed as described (Scholpp et al., 2003). Antisense RNA probes were obtained by in-vitro transcription of full length *axin2* and *lef1* cDNA plasmids.

### Real-time quantitative PCR

Fifty embryos each were lysed in 300 µl TriZol (Sigma) and RNA was prepared using Direct-zol RNA Mini Prep Kit from Zymo Research. cDNA was prepared using MMLV reverse transcriptase from Promega and analysed in a real-time PCR system from LifeTechnologies (ABI StepOnePlus). Primers with the following sequences were used: β-Actin (forward: 5′-CCTTCCTTCCTGGGTATGG-3′, reverse: 5′-GGTCCTTACGGATGTCCAC-3′); Axin2 (forward: 5′-CAATGGACGAAAGGAAAGATCC-3′, reverse: 5′-AGAAGTACGTGACTACCGTC-3′) and Lef1 (forward: 5′-CAGACATTCCCAATTTCTATCC-3′, reverse: 5′-TGTGATGTGAGAACCAACC-3′).

### Western blotting and luciferase reporter SuperTopFlash assay

For luciferase reporter assay embryos were injected with 1.2 ng SuperTopFlash as in (Veeman et al., 2003) and 1.2 ng pCMV-βGal plasmid DNA (gift from Dietmar Gradl) plus synthetic mRNA as indicated. Four pools of ten embryos each were harvested at shield stage and assayed for luciferase activity. The luciferase activity was subsequently normalized to β-galactosidase activity as described. Embryos were harvested in 1% Triton lysis buffer for Wnt luciferase reporter assay and for western blot analysis. Polyclonal antibody against zebrafish Dvl2 (GTX129968 from GeneTex) and polyclonal antibody against human AP2M1 (ab137727 from abcam) were used in a 1∶1000 dilution.

### Microscopy

For *in situ* hybridization and phenotype analysis, embryos were imbedded in 70% Glycerol/PBS (v/v). Images were taken on an Olympus SZX16 microscope equipped with a DP71 digital camera by using Cell A imaging software. For confocal analysis, live embryos were embedded in 0.7% low melting Agarose (Sigma-Aldrich) dissolved in 1×Ringer's solution. Images were obtained by using a Leica TCS SP5 X confocal laser-scanning microscope with 40×or 63×dip-in objectives. Image processing was performed by using an Imaris 6.3.1 software (Bitplane AG).

## Supplementary Material

Supplementary Material
