# Supplementary material for: In vivo analysis of formation and endocytosis of the Wnt/β-Catenin signaling complex in zebrafish embryos
Source: J Cell Sci. 2014 Sep 15;127(18):3970–82. doi: 10.1242/jcs.148767 (PMC4163645; doi:10.1242/jcs.148767)
Supplement: Supplementary Material [file supp_127_18_3970__index.html]

In vivo analysis of formation and endocytosis of the Wnt/β-Catenin signaling complex in zebrafish embryos — Supplementary Material 

# *In vivo* analysis of formation and endocytosis of the Wnt/β-Catenin signaling complex in zebrafish embryos

## JCS148767 Supplementary Material

**Files in this Data Supplement:**

- **Supplementary Material**
