# Supplementary material for: In vivo analysis of formation and endocytosis of the Wnt/β-Catenin signaling complex in zebrafish embryos
Source: J Cell Sci. 2014 Sep 15;127(18):3970–82. doi: 10.1242/jcs.148767 (PMC4163645; doi:10.1242/jcs.148767)
Supplement: Supplementary Material [file supp_127.18.3970_JCS148767.pdf]

Supplementary materials

In-vivo Analysis of Formation and Endocytosis of the Wnt/β-Catenin Signaling Complex in Zebrafish Embryos

Anja I.H. Hagemann[1] Jennifer Kurz[1] Silke Kauffeld[1] Qing Chen[1] Patrick M. Reeves[2] Sabrina Weber[1] Simone Schindler[1,3] Gary Davidson[1] Tomas Kirchhausen[2] Steffen Scholpp[1,*]

[1] Karlsruhe Institute of Technology (KIT), Institute of Toxicology and Genetics (ITG), Karlsruhe, Germany;

[2] Departments of Cell Biology and Pediatrics, Harvard Medical School and Program in Cellular and Molecular Medicine at Boston Children's Hospital, Boston, MA, USA;

[3] Present address: Broad CIRM Center, University of Southern California, Keck School of Medicine, Los Angeles, CA, USA

* Corresponding author : Steffen Scholpp, Karlsruhe Institute of Technology (KIT), Institute of Toxicology and Genetics (ITG), Karlsruhe, Germany; steffen.scholpp@kit.edu

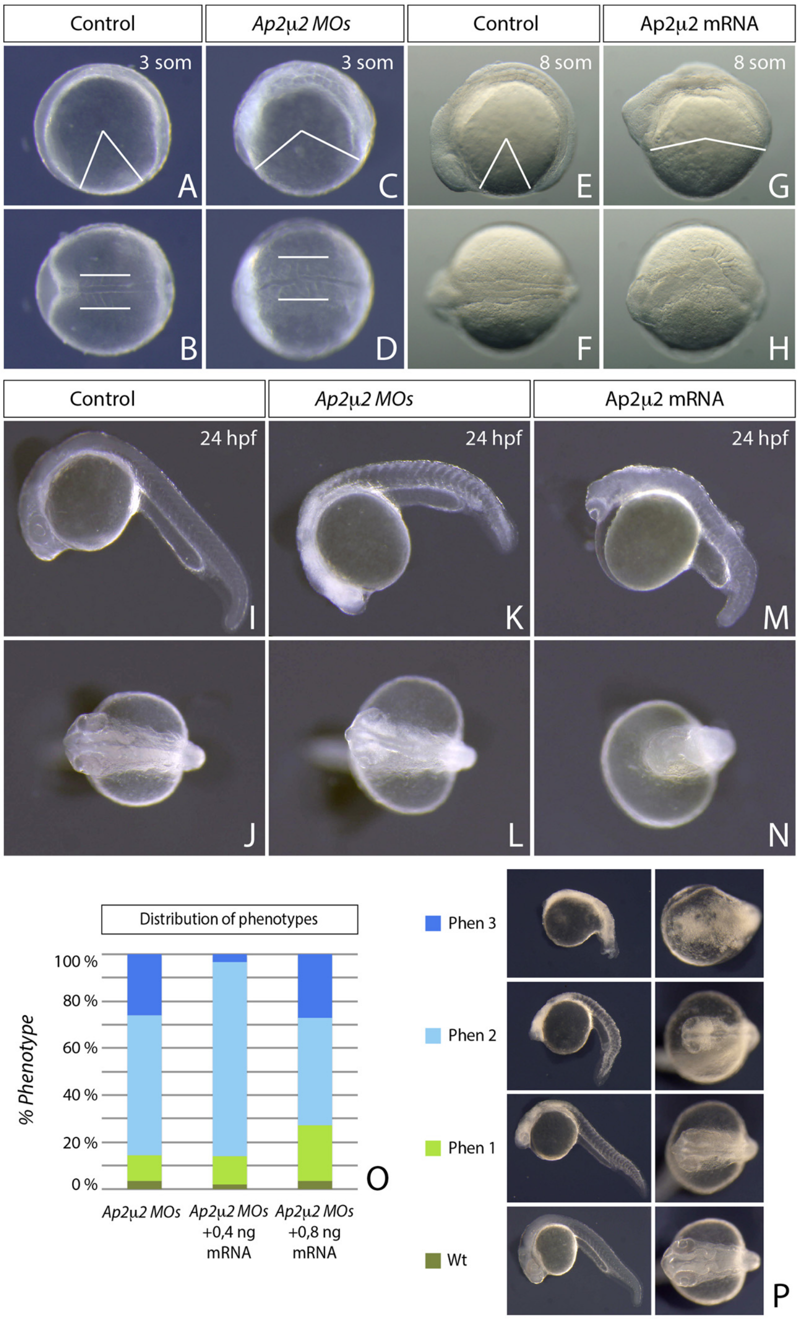

Supplementary Figure 1. Morphological analysis of AP2µ2 function in zebrafish embryogenesis
(A-D, K-L, P) Loss of function analysis of the phenotype at 10 hpf (A-D) or at 24 hpf (I-L, P). (A, B, I, J) Uninjected control embryos. (C, D, K, L) Embryos were injected with 1pmol of ap2µ2 MOs. Injection of either Morpholino alone in one-cell stage embryos did not show any phenotype (data not shown) while double morphant embryos were lethal at late gastrula stage. A reduced Morpholino concentration (1pmol) led to defects in convergence and extension movements during gastrulation (C, D, K, P). Ap2µ2 morphant embryos display a shorter and broader axis. Additionally, embryos showed deformed somites and were severely apoptotic compared to control at 24 hpf (I-L). Heads of 24 hours post fertilization (hpf) embryos were smaller with reduced eye size due to an increase of apoptosis in the anterior brain structures.
(E-H, M, N) Gain of function analysis. Overexpression of Ap2µ2 leads to malformation of the body axis at 13 hpf (G, H) and forebrain structures were reduced with fused eyes at 24 hpf (M, N). In parallel, shorter axes, partially open neural tubes (spina fifida) or neural tube deformations were observed, typical defects in PCP signaling. Hence, we find a combination of Wnt/β-Catenin and PCP phenotypes in both, loss and gain of function analysis during early zebrafish development.
(O,P) Ap2µ2 MOs were injected together with two different concentrations of Ap2µ2 mRNA (O). For statistical analysis, phenotypes were divided into four groups, where mild defects were classified as “phen 1” and strong phenoypes as “phen 3” (P). The main part of the analyzed zebrafish population displayed “phen 2”, small embryos with shorter axes and reduced eyes as well as the previously described white tissue presumably caused by apoptosis. The combination of MOs with 0,4 ng Ap2µ2 mRNA increased the population of “phen 2” in expense of the population with the stronger “phen 3” while the coinjection of 0,8 ng mRNA increased the number of embryos with mild phenotype in expense of “phen 2”. With the higher concentration of mRNA, strong phenotypes were not rescued. This might be explained by an overlapp with the gain of function phenotype, which in the case of Wnt signaling, also leads to developmental defects as mentioned earlier.

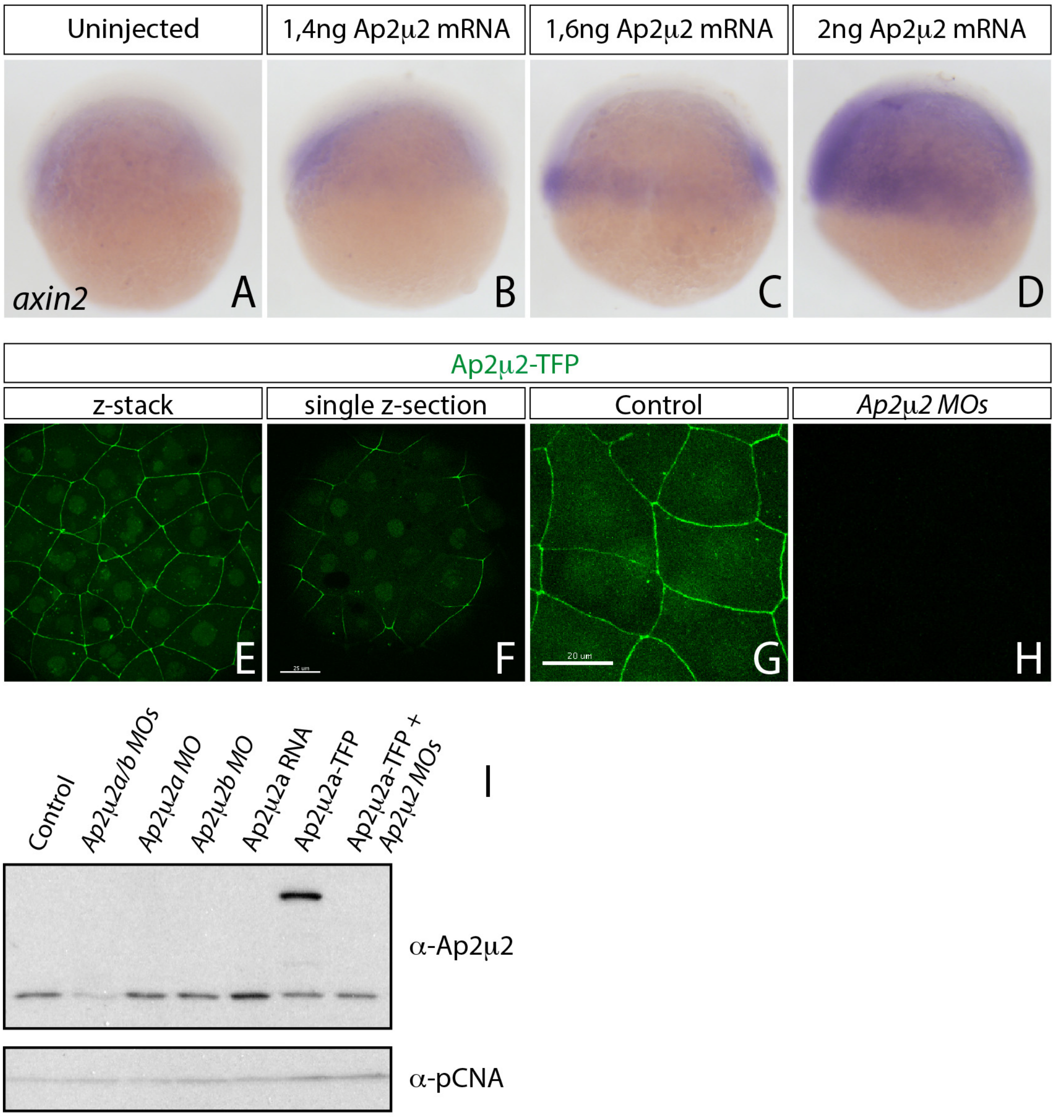

Supplementary Figure 2. Analysis of Ap2μ2 function in Wnt signaling and validation of knock-down approach
(A-D) In-situ hybridization with axin2 probe at shield stage. Embryos are shown from lateral, dorsal to the right. Embryos were previously injected with indicated amounts of Ap2μ2a mRNA. (E-H) Confocal microscopy analysis of live zebrafish embryos previously injectedwith 2ng synthetic mRNA for Ap2μ2-TFP (green). (E) Z-stack of 12μm from animal including epiblast cell layer. (F) Single z-section from (E) cutting through hypoblast. Size bar represents 25μm. (G, H) Z-stacks of 12μm from animal including epiblast cell layer. (G) Ap2μ2-TFP expressing control embryo and (H) co-injected with 1pmol ap2μ2a/b MO each. Settings were unchanged. Sizebar represents 20μm. (I) Western blot analysis of zebrafish Ap2μ2a and Ap2μ2b by a polyclonal antibody against human Ap2μ2 after indicated injections. PCNA was used as loading control. (J-U) Confocal microscopy analysis of live zebrafish embryos expressing indicated mRNAs at 30-50% epiboly stages. Images are the control experiments for Figure 2 and S3, but with single fluorescent channels for better overview. (M, U) show red mCherry signal, (N, O, S, T) show green GFP signal from merges in (J, K, L, P, Q, R).

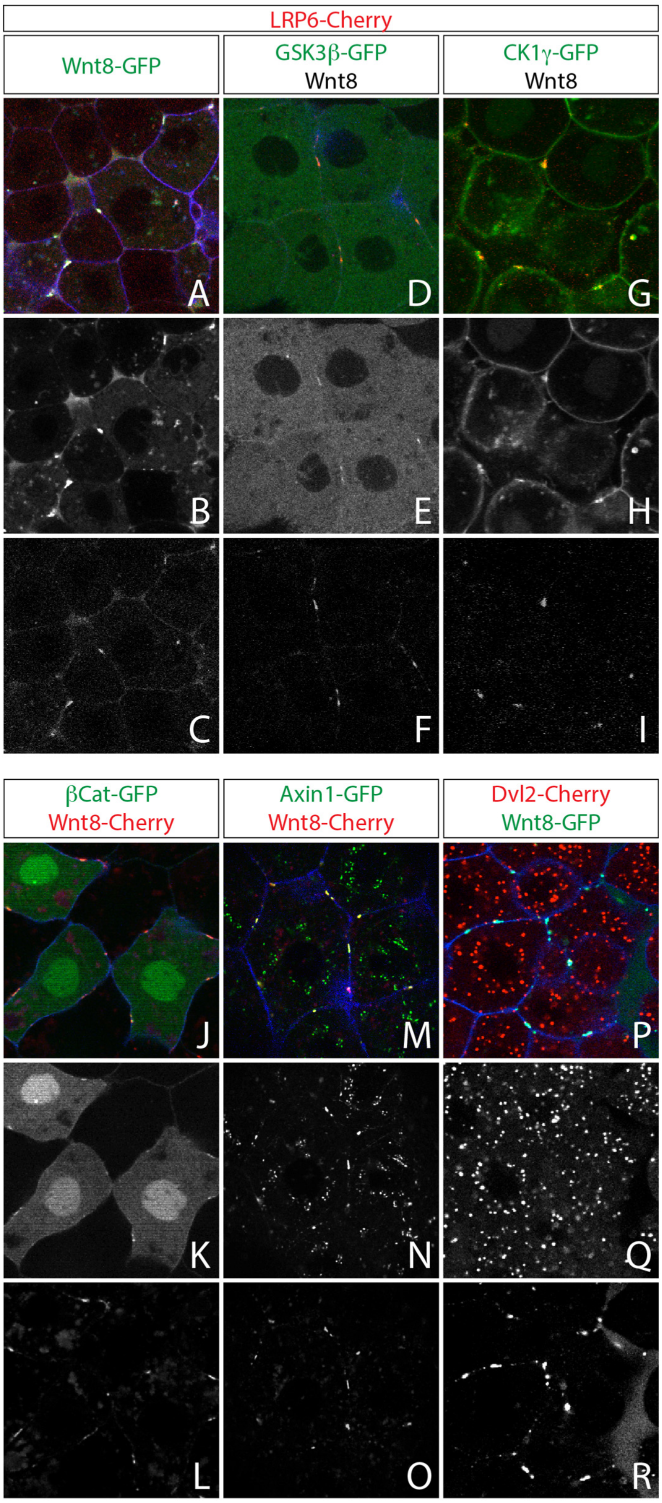

Supplementary Figure 3. Wnt8 induces Lrp6-signalosome formation
Confocal microscopy analysis of live zebrafish embryos expressing indicated mRNAs at 30- 50% epiboly stages. Images are the same as in Figure 2, but with single fluorescent channels for better overview. (B, E, H, K, N and R) show green GFP signal, (C, F, I, L, O and Q) show red mCherry signal from merges in (A, D, G, J, M and P).

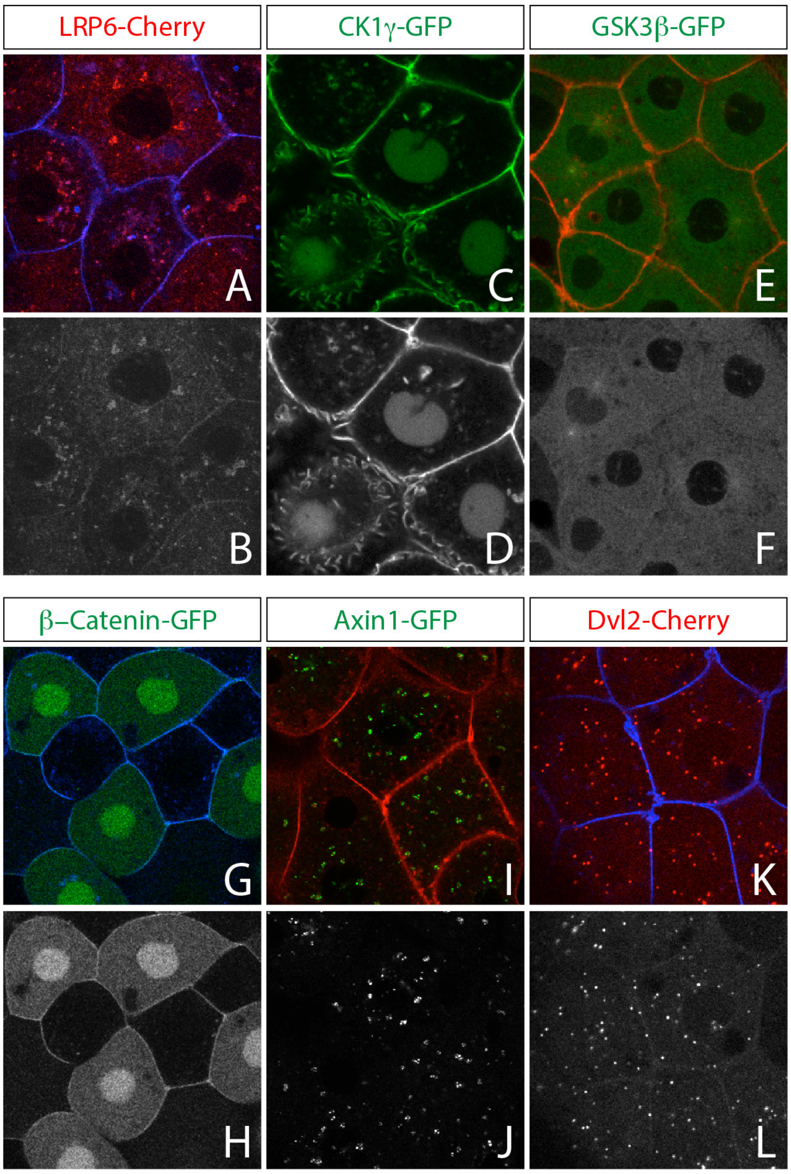

Supplementary Figure 4. Subcellular distribution of members of the transducer complex in the Wnt-off state.
Confocal microscopy analysis of live zebrafish embryos expressing indicated mRNAs at 30- 50% epiboly stages. Images are control pictures to the one in Figure 8 with single fluorescent channels for better overview in addition (B,D,F,H,J,L).

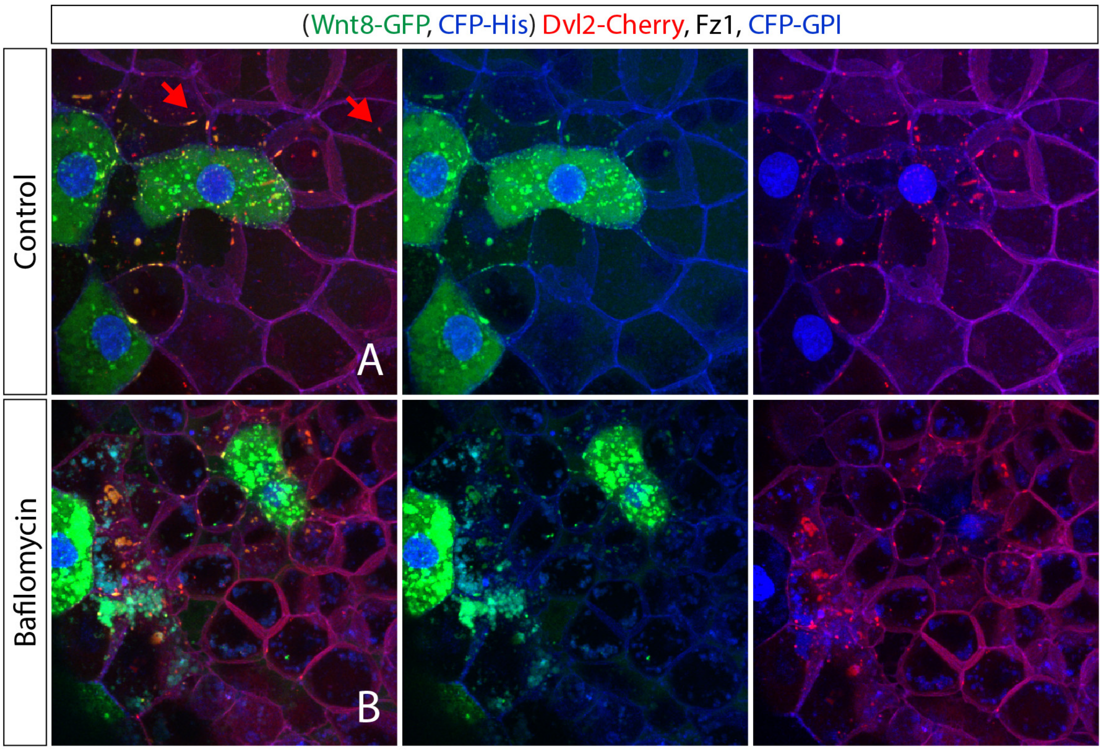

Supplementary Figure 5. Wnt8 induces Dvl2 internalization
Confocal microscopy analysis of live zebrafish embryos expressing indicated mRNAs in a cell clone and in the host tissue at 30-50% epiboly stages. (A) Upon paracrine Wnt stimulation Dvl2 forms aggregates at the cytoplasmic membrane and is internalized in neighboring cells (red arrows). (B) Embryo treated with Bafilomycin for 1 hour before scanning. Host cells display increased localization of Wnt8 and Dvl2. For convenience the Wnt8 producing clones were marked by nuclear CFP. Confocal images represent single z-sections. Constructs were expressed as indicated and are shown in the indicated colors together with membrane markers CFP-GPI (blue).

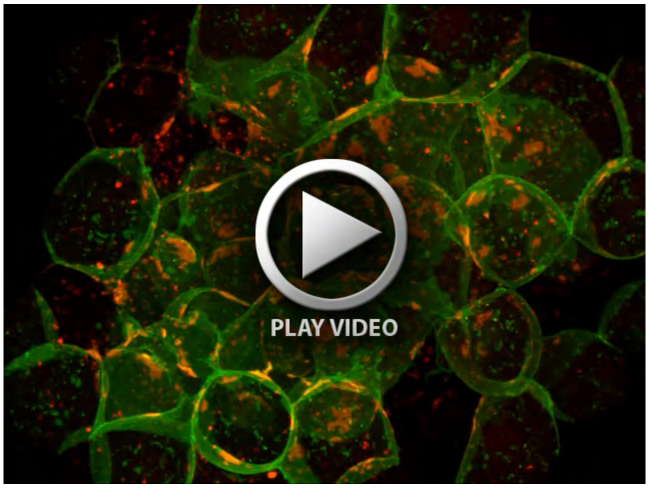

Movie 1. Wnt8 induced Lrp6 signalosomes in zebrafish embryo. 3D animated of z-stack of early gastrula tissue expressing Wnt8, Fz1, Dvl2-Cherry (red) and CFP-GPI membrane marker (green). Dvl2 forms membrane patches upon Wnt8 stimulation.

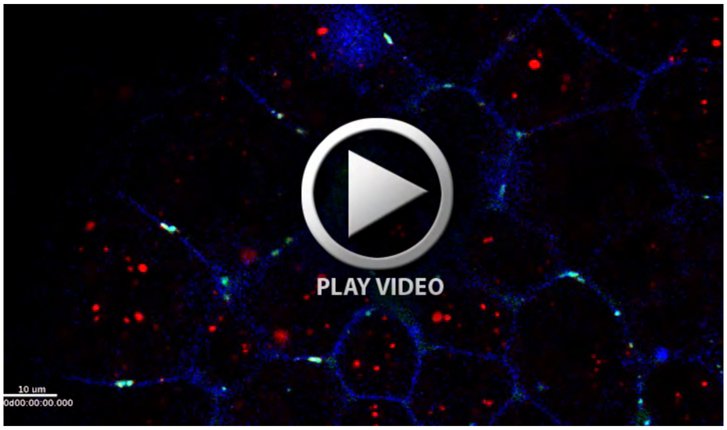

Movie 2. Endocytosis of Wnt8 together with Dvl2 in zebrafish embryo. Endocytosis event of Wnt8 (green) together with Dvl2 (red) from a membrane signalosome. Co-expression of Ap2μ2 and CFP-GPI membrane marker (blue). Movie represents single z-section. Size bar represents 10μm.

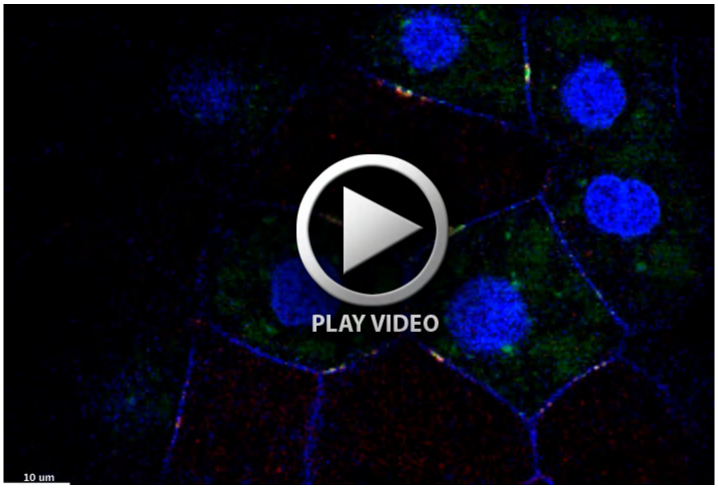

Movie 3. Endocytosis of Lrp6-signalosomes in Wnt8 receiving cells in zebrafish embryo. After paracrine Wnt8 stimulation from clonal cells (marked with CFP-Histone H2B nuclear marker, blue) Wnt8 (green) is internalized together with Dvl2 (red) from a membrane signalosome. Movie represents single z-section. Size bar represents 10μm.
